# English hospital episode data analysis (1998–2018) reveal that the rise in dog bite hospital admissions is driven by adult cases

**DOI:** 10.1038/s41598-021-81527-7

**Published:** 2021-01-19

**Authors:** John S. P. Tulloch, Sara C. Owczarczak-Garstecka, Kate M. Fleming, Roberto Vivancos, Carri Westgarth

**Affiliations:** 1 NIHR Health Protection Research Unit in Emerging and Zoonotic Infections, University of Liverpool, Liverpool, L69 3GL UK; 2 Public Health England, Liverpool, L3 1DS UK; 3 Institute of Infection, Veterinary and Ecological Sciences, University of Liverpool, Liverpool, CH64 7TE UK; 4 Institute of Risk and Uncertainty, University of Liverpool, Liverpool, L69 7ZF UK; 5 Dogs Trust, London, EC1V 7RQ UK; 6 Institute of Population Health, University of Liverpool, Liverpool, L69 3GL UK; 7 NIHR Health Protection Research Unit in Emerging and Zoonotic Infections, Public Health England, Liverpool, L3 1DS UK

**Keywords:** Health care economics, Public health, Trauma

## Abstract

Dog bites are a global health issue that can lead to severe health outcomes. This study aims to describe the incidence and sociodemographics of patients admitted to English National Health Service (NHS) hospitals for dog bites (1998–2018), and to estimate their annual direct health care costs. An analysis of patient level data utilising hospital episode statistics for NHS England, including: temporal trends in annual incidence of admission, Poisson models of the sociodemographic characteristics of admitted patients, and direct health care cost estimates. The incidence of dog bite admissions rose from 6.34 (95%CI 6.12–6.56) in 1998 to 14.99 (95%CI 14.67–15.31) admissions per 100,000 population in 2018, with large geographic variation. The increase was driven by a tripling of incidence in adults. Males had the highest rates of admission in childhood. Females had two peaks in admission, childhood and 35–64 years old. Two percent (2.05%, 95%CI 0.93–3.17) of emergency department attendances resulted in admission. Direct health care costs increased and peaked in the financial year 2017/2018 (admission costs: £25.1 million, emergency attendance costs: £45.7million). Dog bite related hospital admissions have increased solely in adults. Further work exploring human–dog interactions, stratified by demographic factors, is urgently needed to enable the development of appropriate risk reduction intervention strategies.

## Introduction

Dogs have an intrinsic place in modern society with numerous working, health and societal benefits^1–4^. However, as with all animals, they pose an injury risk to humans. Dog bites have been recognised as a global public health issue^5,6^, which can have severe physical^7,8^, infectious^9^ and mental health consequences for humans^10^, and even result in death^11^. They are costly to society in terms of direct^12–14^ (e.g. health care) and indirect^9,15^ (e.g. worker loss, legal and kennelling costs) costs.

The World Health Organisation estimate that dog bites globally lead to ‘tens of millions of injuries’^5^. This is a very crude estimate as no global incidence figures have been calculated, and most countries are lacking incidence data. There has been debate about what the incidence of dog bites in England truly is^6,16^, with claims that medical literature exaggerates the risk^17^. A recent United Kingdom (UK) population-based survey estimated that 25% of individuals have been bitten in their lifetime^16^. A third of those bites required medical treatment, 58.9% of those attended accident and emergency departments (A&E), and only a very small proportion of individuals resulted in hospital admission (1 out of 178 bites); though these were based on a small sample size^16^. Only two analyses of national electronic health records describing dog bites in England have been conducted; both published by NHS Digital (formally Health and Social Care Information Centre)^18,19^. They focus on hospital admissions, in all NHS England hospitals, due to a ‘dog bite or strike’ using Hospital Episode Statistics data, and presented annual increases in absolute case numbers.

The most recent review of hospital admissions figures was based solely on data from the financial year 2014–15^19^. It conducted limited analyses and concluded that the highest incidence of dog injury was found in 0–9 year olds (17.6 admissions per 100,000 population). There was large regional variation, with the highest rates in Merseyside, North-West England, (32.2 admissions per 100,000 population) and the lowest rates were in Kent and Medway, South-East England, (7.3 per 100,000). The rate of admission was 2.6 times higher in the most deprived neighbourhoods compared to the least deprived^19^. These results offer only a static cross-sectional view of limited aspects of hospital records and deliver no insight into temporal trends, and no modelling was performed to explore which demographic variables were associated with dog bite incidence. However, based on the absolute numbers published yearly, without regard for the number in the population at risk, it has been inferred that dog related injuries, interpreted as dog bites, are rising in England^20^.

One attempt has been made to estimate the direct health care costs of dog bites in England^21^. The authors used an unrepresentative sample population (the most and least deprived 10% of the population) from the above report^18^ to estimate the total hospital admissions in 2013, an average cost of a non-elective inpatient stay was applied. They estimated direct costs of dog bite admissions to be about £10 million in 2013. This figure does not include the whole national dog bite admissions population or that attending accident and emergency departments. It is therefore difficult to know how well it reflects the direct health care costs of dog bites in a hospital setting. If the incidence of dog bites is rising, the calculation of improved cost figures is needed so that injury prevention strategies can be justified, and their success measured.

Dog bite prevention strategies have mainly focused on high risk groups, such as children and those that come in contact with dogs through their work (e.g. postal workers)^22^. These interventions are primarily education programmes that focus on interacting with dogs and reading dog body language. The UK government brought the Dangerous Dogs Act 1991 into legislation in order to control dogs that ‘pose a serious danger to the public’, and place restrictions on certain breeds^23^. Despite this legislation and numerous public initiatives designed to reduce dangerous interactions with dogs, dog bite numbers appear to be rising.

Given the belief that dog bites are increasing and have significant public consequences, it is critical to derive accurate incidence figures to support this claim and to understand the demographics of the population affected in order to create effective prevention initiatives. The aim of this study was to analyse English National Health Service (NHS) electronic hospital records to describe the incidence, demographics and flow of dog bite patients in a hospital setting. Using these data, estimates for the annual direct health care cost of dog bites were calculated.

## Results

After removing duplicates, 112,962 FCEs (Finished Consultant Episode; see methods) (107,366 unique patients) were identified with ‘bitten or struck by a dog’ codes, which will now be referred to as ‘dog bite’ admissions, between 1998 and 2018. Ninety-five percent of patients (n = 102,300) were admitted once, 4.3% (n = 4,637) twice, 0.3% (n = 353) three times, and 0.07% (n = 76) more than three times (a maximum of seven times). It is unclear whether these multiple admissions were related to the same dog bite or were multiple bites. The main ICD-10 code given for adults and children was W54.9 (Bitten or struck by a dog—unspecified place; Table 1).

**Table 1:** ICD-10 ‘dog bite’ codes stratified by child–adult status.

ICD-10 code	Description	Number of adult cases	Percentage of named settings	Number of child cases	Percentage of named settings
W54.0	Bitten or struck by dog: home	19,354	80.2	11,570	90.9
W54.1	Bitten or struck by dog: residential institution	64	0.3	3	0.02
W54.2	Bitten or struck by dog: school, other institution and public administrative area	200	0.8	81	0.6
W54.3	Bitten or struck by dog: sports and athletics area	94	0.4	39	0.3
W54.4	Bitten or struck by dog: street and highway	3694	15.3	816	6.4
W54.5	Bitten or struck by dog: trade and service area	533	2.2	136	1.1
W54.6	Bitten or struck by dog: Industrial and construction area	68	0.2	2	0.02
W54.7	Bitten or struck by dog: farm	132	0.5	75	0.6
W54.8	Bitten or struck by dog: other specified places	6965	N/A	1863	N/A
W54.9	Bitten or struck by dog: unspecified place	53,031	N/A	14,067	N/A
Total		84,135		28,652	

### Demographics

**Figure 1:**
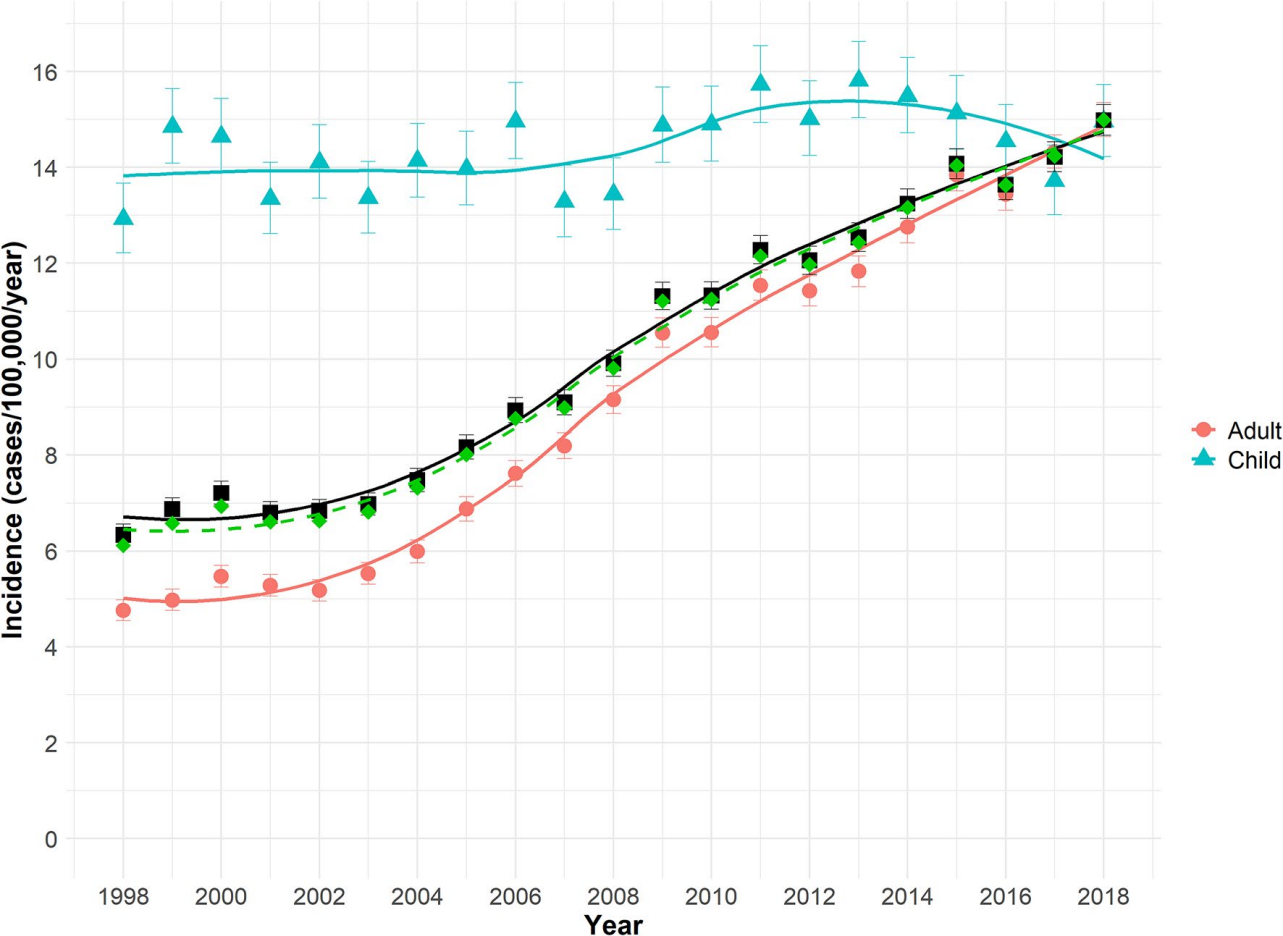
National incidence of dog bite hospital admissions 1998–2018. Crude incidence = black squares, Age-standardised incidence = green diamonds and dashed line.

The incidence of dog bite admissions rose from 6.34 (95% CI 6.12–6.56) admissions per 100,000 population in 1998 to 14.99 (95% CI 14.67–15.31) in 2018 (Fig. 1). Children (14 years or under) made up 25.4% (n = 28,652) of the dog bite admissions. Less than one percent of cases were under one year old (0.5%, n = 595); 43 of these were babies less than a month old, 86 were between one month and six months old, and 466 were between six months and a year old. The incidence of dog bite admissions in children showed no obvious annual trend. The mean annual incidence was 14.44 (95% CI 13.68–15.22) admissions per 100,000 population, with a minimum incidence of 12.93 (95% CI 12.20–13.67) in 1998 and a maximum of 15.82 (95% CI 15.03–16.63) in 2013. In contrast, the incidence of dog bite admissions in adults rose from 4.76 (95% CI 4.55–4.98) in 1998 to 14.99 (95% CI 14.64–15.43) in 2018.

**Figure 2:**
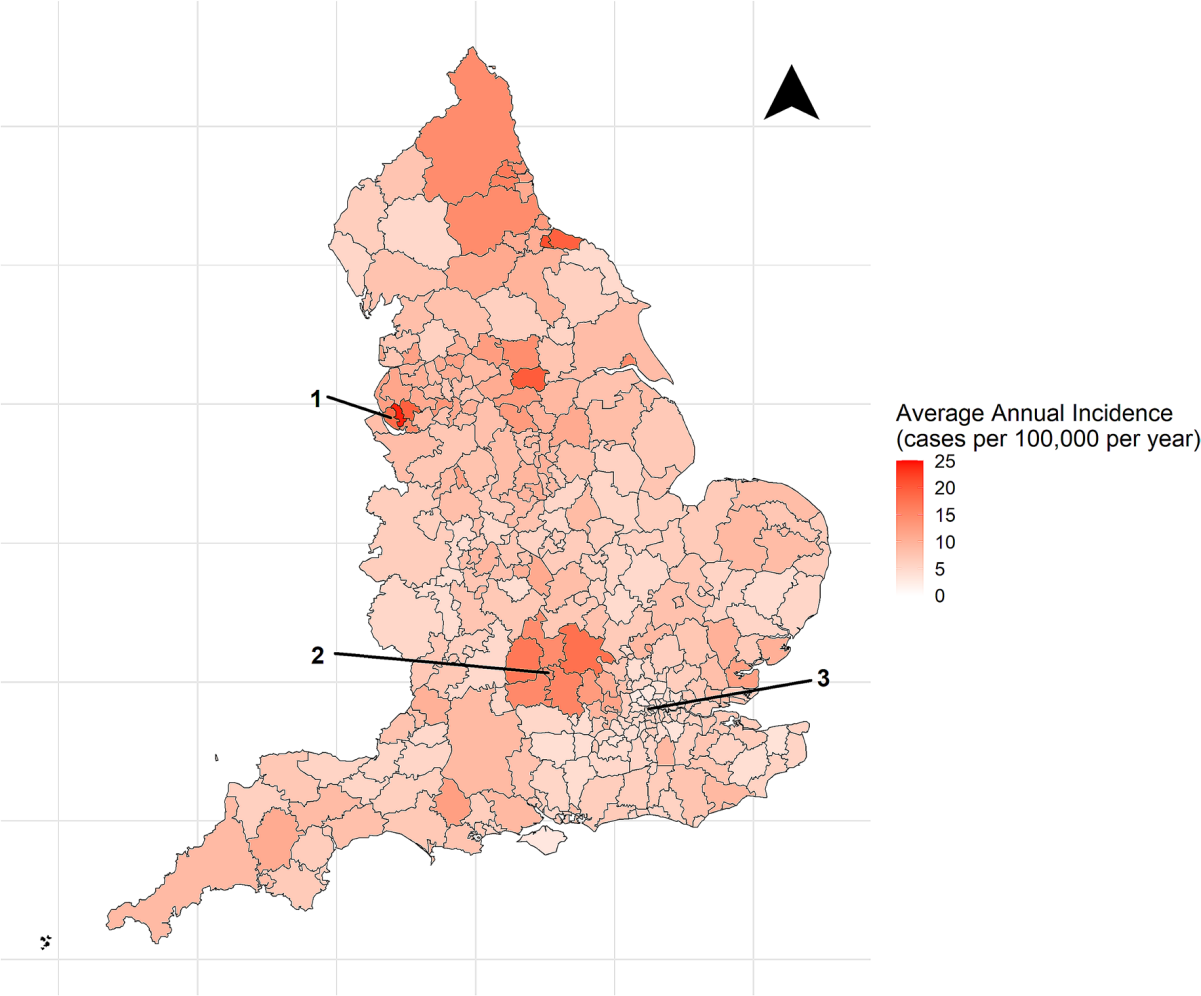
The average annual incidence of dog bite hospital admissions in England (1998–2018) by local authority (1 = Liverpool, 2 = Oxford, 3 = London). (The authors created this map in R (https://www.r-project.org/) using Local Authority boundary shape files created by the Office for National Statistics (Source: Office for National Statistics licensed under the Open Government Licence v.3.0; Contains OS data Crown copyright and database right [2014].)).

The mean annual local authority incidence was 8.0 (95% CI 1.9–14.0) dog bite admissions per 100,000 population per year (Fig. 2). The local authorities with the highest average annual incidence were; Knowsley 24.2 (North-West England), Middlesbrough 21.4 (North-East England), Wakefield 20.0 (North-Central England), Redcar and Cleveland 19.6 (North-East England), and St Helens 19.5 (North-West England). The local authorities with the lowest incidence were; City of London 1.1, Harrow 2.4 (London), Brent 2.7 (London), Barnet 3.0 (London), Isle of Wight 3.1 (South-Central London), and Haringey 3.5 (London).

Almost all FCEs, 99.8% (n = 112,749), had information available about age and sex, 82.7% (n = 93,385) regarding ethnicity, 85.6% (n = 96,686) for IMD decile, and 98.9% (n = 111,717) for rural–urban status. The resultant univariable Poisson regression (Table 2) showed that all the variables explored had significant differences in incidence rate ratios. There was a significant increasing linear trend with year, such that annual admission rates were increasing by 2%. Compared to the national admissions population, males had a higher rate of dog bite admission than females. Age showed a bimodal distribution with the highest rate of admission in children (1–19 year olds, peaking in the 5–9 age group), and the second peak in 40–49 year olds. All ethnicities showed a reduced admission rate compared to the white population, except those patients of mixed race who showed no significant difference in rate. The IMD (Index of Multiple Deprivation; see methods) showed a declining rate of admission as areas became less deprived. Urban areas had a lower rate of admission compared to rural areas.

**Table 2:** Univariate and multivariable Poisson regression analysis for dog bite admission in English hospitals.

Independent variable	n	Univariable analysis		Male multivariable analysis		Female multivariable analysis	
		IRR (95% CI)	p-value	IRR (95% CI)	p-value	IRR (95% CI)	p-value
Intercept		N/A		1.98e−34 (4.35e−36–8.97e−33)	< 0.001	6.28e−41 (1.20e−42–3.25e−39)	< 0.001
**Year (linear)**							
Intercept		1.05e−25 (1.42e−26–7.83e−25)	< 0.001	N/A		N/A	
	112,962	1.02 (1.02–1.03)	< 0.001	1.04 (1.03–1.04)	< 0.001	1.04 (1.04–1.05)	< 0.001
**Sex**							
Intercept		3.91e−4 (3.87e−4–3.94e−4)	< 0.001	N/A		N/A	
Male	57,529	1					
Female	55,389	0.76 (0.75–0.77)	< 0.001	N/A		NA	
**Age (years)**							
Intercept		4.38e−4 (4.27e−4–4.49e−4)	< 0.001	N/A		N/A	
< 1	596	0.07 (0.06–0.08)	< 0.001	0.05 (0.04–0.06)	< 0.001	0.22 (0.19–0.25)	< 0.001
1–4	10,828	2.92 (2.84–3.02)	< 0.001	1.14 (1.09–1.20)	< 0.001	6.94 (6.56–7.34)	< 0.001
5–9	9807	3.77 (3.65–3.89)	< 0.001	1.60 (1.52–1.68)	< 0.001	8.62 (8.14–9.13)	< 0.001
10–14	7421	3.17 (3.07–3.28)	< 0.001	1.63 (1.55–1.72)	< 0.001	5.75 (5.40–6.11)	< 0.001
15–19	5473	1.37 (1.32–1.42)	< 0.001	1.08 (1.02–1.14)	0.007	1.56 (1.46–1.67)	< 0.001
20–24	6362	1		1		1	
25–29	6474	0.83 (0.81–0.86)	< 0.001	0.92 (0.87–0.97)	0.003	0.84 (0.78–0.89)	< 0.001
30–34	6352	0.78 (0.75–0.80)	< 0.001	0.79 (0.75–0.83)	< 0.001	0.85 (0.80–0.91)	< 0.001
35–39	6794	0.94 (0.91–0.98)	0.001	0.69 (0.65–0.73)	< 0.001	1.28 (1.21–1.36)	< 0.001
40–44	7526	1.15 (1.12–1.19)	< 0.001	0.59 (0.55–0.62)	< 0.001	1.91 (1.80–2.02)	< 0.001
45–49	8190	1.18 (1.14–1.22)	< 0.001	0.51 (0.49–0.54)	< 0.001	1.99 (1.88–2.11)	< 0.001
50–54	7823	1.01 (0.98–1.04)	0.64	0.40 (0.38–0.43)	< 0.001	1.78 (1.68–1.89)	< 0.001
55–59	6759	0.78 (0.76–0.81)	< 0.001	0.30 (0.28–0.31)	< 0.001	1.44 (1.35–1.53)	< 0.001
60–64	5826	0.61 (0.59–0.63)	< 0.001	0.21 (0.20–0.22)	< 0.001	1.16 (1.10–1.24)	< 0.001
65–69	5075	0.48 (0.46–0.49)	< 0.001	0.16 (0.15–0.17)	< 0.001	0.92 (0.86–0.98)	0.008
70–74	4159	0.37 (0.35–0.38)	< 0.001	0.13 (0.12–0.14)	< 0.001	0.69 (0.64–0.74)	< 0.001
75–79	3229	0.28 (0.27–0.29)	< 0.001	0.09 (0.08–0.09)	< 0.001	0.57 (0.53–0.61)	< 0.001
80–85	2233	0.22 (0.21–0.23)	< 0.001	0.06 (0.06–0.07)	< 0.001	0.45 (0.41–0.48)	< 0.001
> 85	1860	0.16 (0.15–0.17)	< 0.001	0.05 (0.05–0.06)	< 0.001	0.28 (0.26–0.30)	< 0.001
**Ethnicity**							
Intercept		3.60e−4 (3.57e−4–3.62e−4)	< 0.001	N/A		N/A	
White	88,702	1		1		1	
Asian	1262	0.24 (0.23–0.25)	< 0.001	0.22 (0.20–0.24)	< 0.001	0.10 (0.09–0.11)	< 0.001
Black	1102	0.38 (0.36–0.41)	< 0.001	0.39 (0.36–0.42)	< 0.001	0.17 (0.15–0.19)	< 0.001
Chinese	88	0.33 (0.27–0.40)	< 0.001	0.25 (0.17–0.35)	< 0.001	0.28 (0.20–0.38)	< 0.001
Mixed	919	1.01 (0.94–1.07)	0.88	0.68 (0.62–0.64)	< 0.001	0.59 (0.53–0.65)	< 0.001
Other ethnic group	1312	0.76 (0.72–0.80)	< 0.001	0.59 (0.54–0.64)	< 0.001	0.48 (0.44–0.53)	< 0.001
**Index of multiple deprivation (linear-starting at 1)**							
Intercept		4.58e−4 (4.52e−4–4.64e−4)	< 0.001	N/A		N/A	
	96,686	0.94 (0.94–0.94)	< 0.001	0.91 (0.91–0.92)	< 0.001	0.96 (0.95–0.96)	< 0.001
**Rural urban**							
Intercept		3.88e−4 (3.83e−4–3.93e−4)	< 0.001	N/A		N/A	
Rural	23,264	1		1		1	
Urban	88,453	0.87 (0.86–0.88)	< 0.001	0.81 (0.79–0.84)	< 0.001	0.73 (0.72–0.75)	< 0.001

*IRR* incidence rate ratio, *CI* confidence interval.

As age and sex often interact with each other, two multivariable models were created, one for each sex. This additionally provides clear sex-disaggregated data, as encouraged by the World Health Organisation. The male model only used male admissions for the denominator, and the female model only used female admissions. Both models showed a significant increase of admission rate, 4% annually. In the male model, the highest rates of admission were in children and young adults (1–19 year olds) and reached their peak in 10–14 years old. From 25 years onwards, the rate of dog bite admission declined with age. All ethnicities had a significantly lower rate of dog bite admission compared to those who identified with being white. Both models showed similar trends in IMD and rural–urban status to that shown in univariable analysis. However, the female model showed a larger difference in admission rate between rural and urban areas. Females showed the same trends in all variables except age. The results showed two female age groups with high rates, one between the ages of 1–19, and a second between 35 and 64; the first group peaked with 5–9 year olds and the second at 45–49. After 65–69 years old the rate of admission declined. The age groups with the lowest rates of admission for both male and females were the less than one year olds and the greater than 85s. Both models showed small residual differences and proved to have good model fits; both had p = 1. Due to this, no further model diagnostic evaluation was performed.

### Accident and emergency attendance estimates

In the A&E dataset only 11 hospitals supplied data, 6.5% of all English NHS hospitals (Acute Trusts, n = 168^24^), which contained dog bite codes. Only 5,772 patients were coded with a dog bite between 2008 and 2017. A weighted A&E admission rate of 2.05% (95% CI 0.93–3.17) was calculated. Through triangulation with the more robust admissions data, the weighted rate was used to estimate the overall number of A&E attendances for a dog bite in England. In the admissions data, a total of 89,158 patients were recorded as being admitted through A&E; if 2.05% of patients who attend A&E for dog bites get admitted then 4,349,171 (95% CI 2,812,555–9,586,882) A&E attendances may have occurred in England between 1998 and 2018. This represents an average of 207,103 (95% 133,931–456,518) A&E attendances per year.

### Direct health care cost estimates

**Figure 3:**
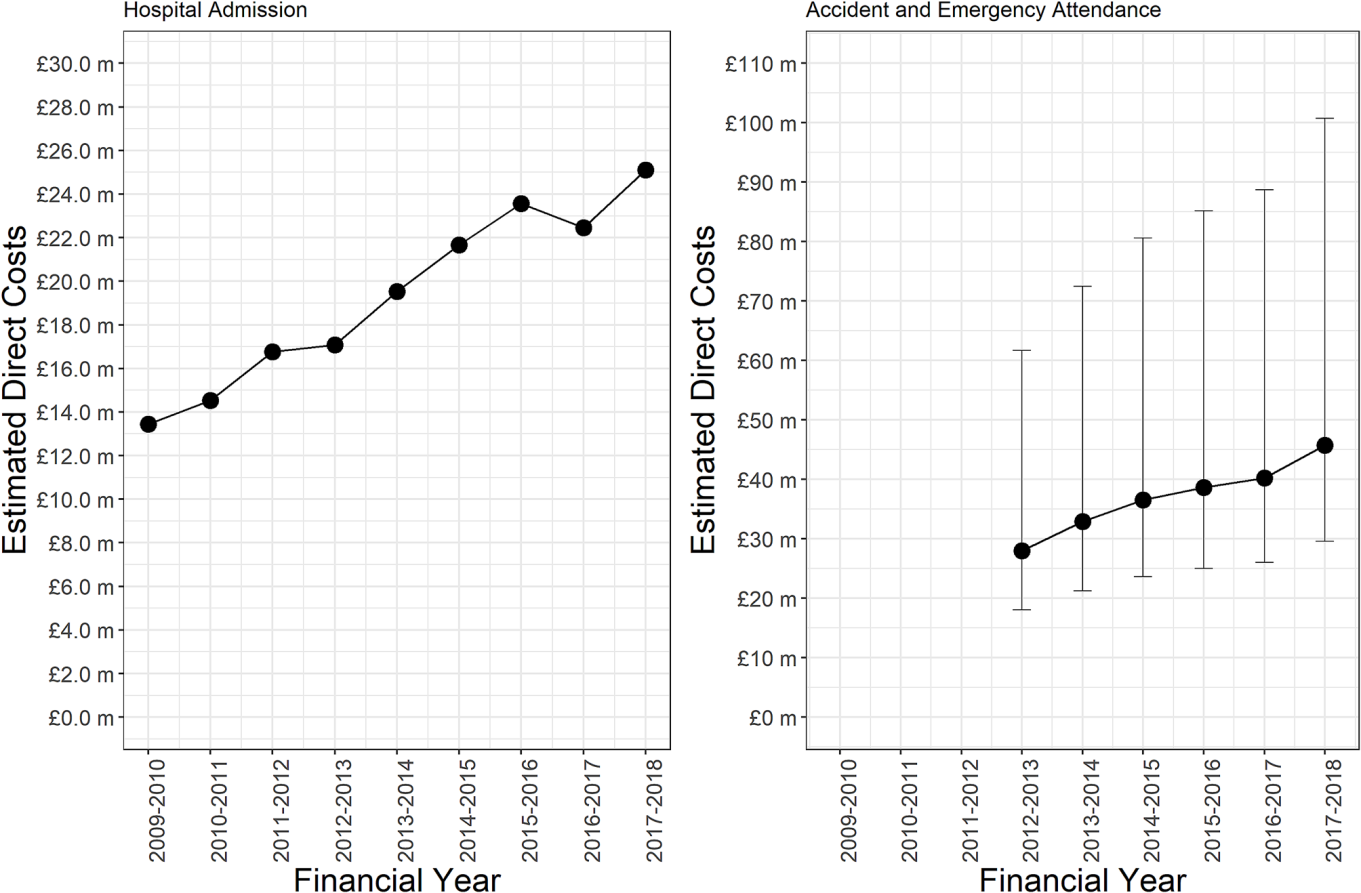
Estimated direct health care costs of dog bite hospital admissions and accident and emergence attendance in England.

Between the financial years 2009/2010 and 2017/2018 the total estimated direct costs of dog bite admissions were £174,188,443. There was a significant rise in costs (p < 0.001, adjusted r2 = 0.96), the lowest year being 2009–2010 (£13,450,820) and the highest being 2017/18 (£25,114,772) (Fig. 3). Confidence intervals could not be calculated as both component parts of the cost estimate, case numbers and unit costs, did not have population parameters associated with them.

Between the financial years 2012/2013 and 2017/2018 the total estimated direct costs of dog bite A&E attendances were £222,041,073 (95% CI £143,591,230—£489,445,376). There was a significant rise in costs (p < 0.001, adjusted r2 = 0.96), the lowest year being 2012/2013 (£27,970,244; 95% CI £18,088,013—£61,654,839) and the highest being 2017/2018 (£45,713,171; 95% CI £29,562,145—£100,765,591).

## Discussion

Dog bites are a growing public health problem which is costly to society. This work has identified an increase in the incidence of hospital admissions in England due to dog bites and a doubling of incidence over 20 years. This is the first study to identify that this rise has been driven by an increasing number of adults being admitted, whilst rates in children have remained relatively static. Males had higher admission rates, whilst the age groups with the highest relative rates of admission were children between the ages of 1 and 19, and women between the ages of 35 and 64. Admission rates were significantly higher in those of white ethnicity, and in rural areas compared to urban areas. The most deprived neighbourhoods in the country had the highest incidence of bites. The map produced is the highest resolution of dog bite data to date and shows large geographical variation between local authorities. Recorded deaths equated to roughly four dog bite related deaths a year, likely an underestimate as it only includes individuals who have died at hospital. The number of children under 1 year of age, and in particular under one month of age (n = 43), that were bitten is highly concerning. In the financial year 2017/2018, dog bite hospital attendance and admissions may have cost the NHS £70,827,943.

### Strengths of study

This is the first longitudinal analysis of UK dog bites. Trends have been identified that were unknown due to the cross-sectional nature of prior research, principally, the incidence of adult bites has tripled in twenty years and that of children has stayed stable but high. The time scale and size of these data enable greater confidence in describing the demographics of dog bite victims who present to hospitals. The patient management data provides a detailed classification of the resultant injuries from dog bites and their severity (Supplementary Material). This is the first time that dog bite costing estimates have been calculated for consecutive years for both hospital admissions and A&E attendance.

### Limitations

The accuracy of studies based on HES are reliant on two things: the specific ICD-10 codes used to build case definitions and the quality of the clinical coding performed by the hospitals providing the data. The validity and quality of clinical coding in HES has been much discussed and there is inherent variability in coding standards between individual coders and hospitals^25,26^. The degree and the direction in which this bias the results is unknown. HES coding is based on the patients written discharge summary and coders therefore rely on the quality and level of details in this summary for their choice of subsequent codes^27^. No consensus has been reached over the degree of coding accuracy and improvement, but there are suggestions that financial incentives may have improved coding quality in recent years. In terms of geographical recording, data is based on the patient’s home address. Therefore, a degree of error in mapping incidence may occur if the patient is bitten away from their household, such as a delivery worker. However, we believe that this error is likely to be small as the majority of bites are recorded as occurring in the patient’s home. The ICD-10 codes used in this study produce another problem as they are defined as ‘bitten or struck by dog’. These results will overestimate the number of dog bites as they include any dog-related injury^6^. Despite this, we have confidence that the results presented here are largely representative of dog bite patients due to the stratification of the patients by their injury type (Supplementary Material, Table S1). Non-bite dog-related injuries in children predominately present as abrasions, lacerations and fractures^28^, and a maximum of 4.15% of children injured by dogs fell into these injury type classifications. Dog bite injuries to adults predominately describe lacerations, open wounds and superficial injuries^7,29–31^, which make up 77.5% of the injuries in these data, so they are again likely to represent bites. Some of the remaining injuries, such as traumatic amputation (2.9%) are highly likely to be associated with dog bites, whilst others, fractures (13.2%), could be a result of any type of dog-related injury. However, without accessing the written medical notes of each patient there is no way of knowing what type of dog-related injury has occurred.

The second limitation concerns direct cost estimations. The ICD-10 codes used to identify dog bite admissions do not have an associated NHS direct health care cost. They are all ‘causal’ codes rather than ‘diagnostic’ or ‘procedural’ for which costs are available. This meant that we had to use a proxy unit cost. For admitted patients we used the average unit cost of a ‘Non-elective inpatient admission’; for the financial year 2017/2018 this equated to £3117 per admission. Unfortunately, as discussed above, no confidence intervals could be calculated so we could only present single point estimates. Caution must therefore be taken interpreting our crude costs as we do not know the limits of the range in which the true cost lies. Secondly, the unit cost is based on the average unit cost of a type of admission that contains a huge variety of clinical presentations or procedures. In 2017/18 a unit cost ranged from £75 to £129,802^32^. As dog bites have a variety of clinical manifestations, and with no dog bite specific unit cost, it is difficult to know how representative this average cost is for dog bite injuries. Considering that many severe dog bites require extensive reconstructive or orthopaedic surgery^7,13,33^, we believe that it is likely that these costs underestimate the true cost. A full cost assessment of each case is needed to be able to provide more accurate direct healthcare costs of dog bite admissions.

The methodology to estimate the number of cases attending A&E is crude. A&E data quality is notoriously poor in HES^25,26,34,35^; in our study only 6.5% of hospitals provided data. How representative these hospitals are is unknown, and this places bias on our attendance calculations. That our calculated admission rate is similar to other nations’ estimates gives credence to our figures^8,36^. Our extrapolative methodology and small sample size results in wide confidence intervals for the subsequent estimate of A&E attendance and associated direct health care costs. The unit cost for these calculations is purely the average cost of an attendance to A&E and does not include any treatment or management costs of the patient, so is likely an underestimate. The confidence intervals are understandably, and necessarily, wide and a high degree of caution must be taken interpreting or implementing actions based solely on these costs. New studies are needed to evaluate the burden that dog bites place on A&E departments, describe the clinical presentations of cases, and to calculate more accurate direct cost estimates of dog bites. The new Emergency Care Data Set has the potential to explore this further^37^.

There are other healthcare costs that could not be calculated by this research. The outpatient data was exceptionally sparse and so we have no understanding of the burden, demographics or costs associated with hospital outpatient departments, nor in primary care or in other health care settings such as walk-in centres. We have additionally not focused on indirect healthcare costs, such as time-off work, worker replacement, changes in productivity, and long-term morbidity (including mental health issues).

### Comparisons to existing literature

There are striking similarities to previous research; most countries describe children having the highest incidence of dog bites^8,14,30,36,38–40^ which was seen here until 2017. Alongside other high-income countries, England has seen an increase in hospital dog bite admission. The current incidence of 14.99 cases per 100,000 in 2018 is higher than many other high income countries (12.39 in Australia^41^, 1.5 in the Netherlands^36^), but lower than the USA, which still appears to have the highest incidence (110 cases per 100,000 per year^8^). There are many societal and healthcare differences between these nations, but these data suggest that England is on the higher end of the spectrum concerning the number of annual dog bites. However, the overall number of dog bites in England is likely to be much higher than the level described here. Only the most seriously injured patients will be admitted into hospital, as evidenced by the injuries described in the Supplementary Material. Those that attend primary care, self-treat, or do nothing will not have been captured by our data. As mentioned, it has been reported that only a small proportion of dog bites result in hospital admission^16^. Other papers acknowledge that hospital data only provide limited information on the wider dog bite public health problem^8,41,42^. Research in a variety of health care and community settings is needed to understand the true extent of the issue.

The reasons for the rise in dog bites cannot be ascertained from this data alone but a number of speculations can be made about what might have changed. To the authors’ knowledge, only one other paper, describing hospitalisation in Australia, mentions an increase in dog bites admissions being driven by an increase in adult admissions, whilst child admissions remain stable^41^. However, Australian adult incidence never reaches parity to that of children. From these data it is unclear what is driving this increase in adults being bitten by dogs, and why the only adult group showing an increase in admission rate is 35–64 year old women. It could be due to differences in health-seeking behaviour in different age groups and sexes. However, our Poisson models use the entire hospital admissions data as the denominator population and so excludes this as an explanatory reason.

One plausible explanation of the increasing number of dog bites is greater exposure due to increasing number of dogs. The estimated UK dog population has risen from 7.9 million in 2010 to 9 million in 2018^43^. There have been changes in pedigree breed preferences which have been theorised to influence dog bites; small breed types have increased in popularity^44^. However, given the specificity of rises in bites to adults, numbers of dogs or breeds owned is unlikely to be a causal factor. Further, there is no clear evidence that bite risk is associated with breed^45,46^ despite the continued perception, and legislation^23^ suggesting that it does^47^.

Changes in how dogs are sourced, or how we interact with them, may also be theorised to impact bite incidence. The number of dogs that are moving across borders through the Pet Travel Scheme has increased dramatically from 85,000 in 2011 to over 275,000 in 2016^48,49^. Many are commercially bred puppies who may miss out on appropriate socialisation and experience the lengthy transport as distressful, which may impact on their behaviour later in life^50–52^. Commercially bred dogs are also more likely to have behavioural issues compared to non-commercial breeders^53,54^. For example, they are three times more likely to show owner-directed aggression, and 1.6 times more likely to show stranger-directed aggression. Dog owners report having an anthropomorphic relationship with their dogs^55^, and these relationships are resulting in new expressions of love and care for their pet^56^. Unintentionally, these changes may lead to conflict in human–dog interactions, increasing the chances of aggressive behaviours. Currently, 53% of dogs of a given breed do not meet their exercise guidelines^57^, and 24% percent of dogs are left alone at home every day for more than five hours^58^. This may deprive them of adequate social contact and also induce frustration. These ideas are all speculative as no study has linked the above potential risk factors to an increase in dog bites. It is unlikely that the rise in dog bites is due to an increased inherent risk of aggression posed by the actual dogs involved (such as socialisation levels or source) as there is not a feasible explanation why this risk would differ so dramatically between ages of the victim.

Further work is needed to define what is driving the increase in dog bites in England, and specifically to adults. Differences in dog ownership patterns could be a possibility; if the increase in dog numbers vary between age strata and household type (i.e. young family, single occupancy, retired couple) then specific populations more at risk may have changed over time. It could be hypothesised that rising dog bites are due to an increase in home postal deliveries. Previous research has shown that delivery workers are more frequently bitten compared to other professionals, but their demographics are predominately middle-aged men^29^. Our data show the majority of bites in adults occur at home (80.2%), and the main demographic with an increase are middle-aged women. It is therefore unlikely that this is the sole explanatory cause for an increase in incidence. A final scenario could be that dog bite intervention programmes, which are predominately aimed at children and those who are exposed to dogs at work^22^, have been so successful that they have helped to maintain the incidence of dog bites in these high risk groups despite an overwhelming background increase in incidence. Further research is required to understand the causes of these data patterns, but a potential implication is that future prevention strategies should include older demographics.

Many studies describe a predominance of dog bites in men across all age groups^8,16,40,41^. Univariable analysis showed a higher admission rate for men than women. In the male multivariable model, the highest rates of admission were in children with a decline in admission rates from 30 years old onwards. Conversely, the female model displayed admission rate trends that appear to be unique. The initial peak in children is similar to previous studies, however we can find no other literature describing a second admission peak in women between the ages of 35 and 64. This demographic needs to be explored to understand whether there are any behaviours or interactions that make predominately middle aged women more susceptible to being bitten and admitted to hospital.

Our work is the first to show detailed stratification of dog bite admission based on ethnicity. It is interesting that both male and female models show the same differences between ethnicity and admission rates, with ‘white’ patients having the highest rates of admission. This may be due to cultural differences in ownership and interactions with dogs. For example, in a Liverpool focused study, the area with the highest incidence of dog bites in England, ‘non-white’ children were 0.23 times less likely to own dogs than ‘white’ children^59^.

The geography of patients’ resident location is complex and challenging to interpret. The patients neighborhood deprivation status was correlated with a higher incidence of bites, which supports previous cross-sectional analysis of HES^18^. Factors typically correlated with higher levels of deprivation have been found to be better predictors of hospital admissions due to bites than any demographic variable^60^. Some of the areas with the highest incidence of dog bite admission, such as Merseyside (North-West England) and Wakefield (North-Central England), have generally high levels of deprivation. However, there were significant anomalies. Oxfordshire (South-Central England) has some of the highest incidence of dog bite admissions (Aylesbury Vale 17.8 admissions per 100,000 per year, West Oxfordshire 17.0) but is one of the least deprived areas, and Greater London has some of the most deprived areas but has some of the lowest incidence of admissions. Differences in dog population do not entirely explain these results as the areas with the largest dog populations, the North-West and South-East of England^43^, have some of the highest and lowest incidence of admissions respectively. Rural–urban status, likewise, does not give a logical explanation. Through our Poisson model, we have shown that English dog bite admissions are similar to other nations and are higher in rural areas^40,42^. This challenges previous work that described no differences in English dog bite admission numbers due to rural–urban status^19^. These results highlight that the risk factors associated with dog bite admission geography, rural–urban status, and deprivation is likely to be multifactorial and research is needed to disentangle this.

The majority cases were admitted through accident and emergency departments. An American study estimated that there were 337,103 dog bite emergency departments attendances annually making up 1.1% of all attendances^8^. In comparison, the average annual number of dog bite attendances estimated for England was 206,980, this would equate to 0.8% of all attendances^61^. In the USA, 1.7% of dog bite emergency attendances lead to hospital admission^8^, 2.7% in the Netherlands^36^, whilst in England this was estimated to be 2.1%. The variation in the degree of healthcare privatisation between the USA, the Netherlands and England mean that the estimates calculated here are not completely comparable. However, they do suggest that the estimates calculated within this paper are reasonable and need exploring with a more robust methodology. Our direct health care costings are an improvement on previous research methodologies^21^. Further inspection of hospital records, at a national and individual trust level, is needed to understand how dog bite victims are managed elsewhere within the NHS systems. Further work is needed to calculate and model more accurate direct and indirect health care costs across a variety of different health care settings before we can understand the true cost of dog bites to England.

## Conclusions

The incidence of dog bites in children has stayed consistently high over twenty years, whilst incidence in adults has tripled. Despite sustained education and preventative campaigns across large parts of society, the issue of dog bites continues to grow. Clinicians are at the forefront of this ever-growing problem and have raised concerns that the root of this public health issue has not been addressed. Legislation around breed types^23^ is unlikely to solve this issue as dog bite risk has been shown to be complex and multifactorial. Research is required to develop new effective intervention strategies in response to the changing demographics of bite victims, so that the risks of living and working with dogs can be minimised and the benefits fully realised.

## Methods

### Data collection

Hospital Episode Statistics (HES) collates data into datasets that contain information about (1) admissions, (2) A&E attendances and (3) outpatient appointments, in National Health Service (NHS) hospitals in England^62^. These data have been used for the calculation of health care costs and are mainly administrative in nature. Records within A&E and outpatients datasets are often incomplete with inconsistent recording^34^. A preliminary query of the outpatients’ dataset only returned 35 records for dog bites, and 29 of these were from the same outpatient department. Due to biases inherent in their small numbers and lack of representativeness, outpatients’ data were excluded from our analyses. The admissions dataset is the most robust and has been used regularly for epidemiological research^63^; therefore this paper will principally focus on the admissions dataset.

Access to the HES database was provided through a data access agreement between Public Health England (PHE) and NHS Digital. Data were provided in a pseudo-anonymised format. We identified finished consultant episodes (FCE) in which patients were coded with a ‘dog bite or strike’ according to the International Statistical Classification of Diseases and Related Health Problems 10^th^ Revision (ICD-10)^64^ (Table 1). A finished consultant episode is the analysable unit of HES and refers to the time a patient spends under continuous care from admission to the point of discharge or death. As previously highlighted^6^, this definition based on ICD-10 codes does include other dog-related injuries. The proportion directly related to dog bites is unknown and unidentifiable through the analysis of national hospital electronic health records. The impact of this will be discussed.

Data were extracted for patients presenting between 1 January 1998 and 31 December 2018 who had a dog bite code in any of the ‘external cause’ fields in the HES admissions dataset. ‘Dog bite’ codes are not placed in any of the diagnosis fields of HES. These fields describe the nature of the resultant injury that has occurred and were analysed separately (see Supplementary Material). Patient level variables examined included the injury setting (based on the ICD-10 codes in Table 1), sex, age, ethnicity, and the anatomical location and pathology resultant of the injury. Data regarding patient geography was also examined, including; local authority of residence, rural–urban status and the index of multiple deprivation (IMD) decile^65^. The IMD measure the relative levels of deprivation in 32,844 small areas in England; each area contains between 400 and 1200 households. IMD is comprised of seven weighted domains which are combined to give an overall score and subsequent rank^65^. These include: income, employment, health deprivation and disability, education and skills training, crime, barriers to housing and services, and the living environment. For routine HES analysis the IMD ranked areas are then placed into deciles. The first IMD decile contains the 10% most deprived neighbourhoods in England, whilst the tenth decile contains the 10% least deprived. Rural–urban status is defined by the Office of National Statistics, and is applied to the same small area geographies used to define IMD^66^. The definition is based upon both population size and population sparsity in the surrounding geographies. Note these geographical variables all relate to the area of the patient’s residence, not that of the hospital.

### Incidence and demographic analysis

The annual incidence of dog bite admissions for England was calculated and stratified by child–adult status, using the Office for National Statistics (ONS) mid-year population estimates as the denominator population^67^. Due to the age-bands used by the ONS, a child was defined as being less than or equal to 14 years of age. Cases between 15 and 18 could not be defined as children as they sit within the 15 to 19 age band, which contains adults; national denominator data could not be presented at a higher resolution. Alongside the crude annual incidence, an age-standardised incidence was calculated via direct standardisation with the 2013 European Standard Population^68^. The average annual incidence in each local authority was calculated and plotted on a map; this was based on the patient’s residence rather than where they were bitten.

Using the identified cases and the national HES admissions population as the denominator, we assessed the following variables using Poisson regression; year, sex, age, ethnicity, IMD and rural–urban status. Any variables that were found to be significant with univariable analysis were taken forward for multivariable analysis. The age-band of 20–24 years old was chosen as the reference age band in analysis as this was likely to be representative of the healthy adult population. Goodness-of-fit Chi-squared tests for Poisson models were performed on all multivariable models created to assess overall model performance. If there was a poor model fit, then overdispersion diagnostics would be performed.

Methodology and results describing bite setting, resultant injury, and patient management are compiled in the Supplementary Material.

### Accident and emergency attendance estimates

Data from the HES A&E dataset were extracted where the ‘diagnosis’ field included a dog bite ICD-10 code. HES A&E data has known issues for injury data. To improve speed of coding and reduction of clinical burden, at the time of the study, clinicians were encouraged to code solely for injury type, a broad cause of injury, and anatomical location^62^. A dog bite fits under the injury type of ‘Bites/Stings’. Clinicians were under no obligation to define it further to a dog bite, we therefore expect large coding gaps in the data. Recently NHS England has adopted a new A&E dataset and coding nomenclature that may resolve these issues. For each department reporting dog bites, the percentage of patients admitted to the hospital was calculated. A weighted mean admittance rate was calculated; weighting was based on the number of patients attending A&E for a dog bite for that hospital. This figure was applied to the total number of patients being admitted to all English hospitals, as recorded by the admissions data, to estimate the total number of attendances to A&E for the study period.

### Direct health care cost estimates

Crude estimates of direct health care costs were calculated by multiplying the annual number of FCEs, in a financial year, by the annual average unit cost of a ‘Non-elective inpatient admission.’ This is defined as an ‘admitted patient care activity which takes place in a hospital setting where the admission was an emergency/non-elective’^32,69^. This unit cost was chosen as the majority of cases were admitted through A&E and would therefore be classified in this admission category. Total costs were only presented for the financial years 2009/2010 through to 2017/2018 as they had consistent cost definitions, unlike the remaining years. To estimate the direct health care cost of dog bites in A&E, the estimated number of A&E attendances for each financial year were multiplied by the annual average unit cost of ‘Accident and Emergency Attendance’^32^. Consistent cost definitions were only available for financial years 2012/2013 through to 2017/2018. Trends in cost over time were tested for significance with linear regression.

All statistical and spatial analyses were carried out using R language (version 3.2.0) (R Core Team 2015). Results were deemed statistically significant where p < 0.05.

### Ethics approval and consent to participate

No ethical approval was required as these data were collected for public health surveillance under The Health Protection Legislation (England) Guidance 2010^70^. Hospital Episode Statistics (HES) data were made available by NHS Digital (Copyright 2015, re-used with the permission of NHS Digital. All rights reserved.) Approvals for the use of anonymised HES data were obtained as part of the standard NHS Digital data access process.

## Supplementary Information

Supplementary Figure S1.

Supplementary Information.

## Data Availability

The data governance arrangements for the study do not allow us to redistribute HES data to other parties. Researchers interested in accessing HES data can apply for access through NHS Digital’s Data Access Request Service (DARS) https://dataaccessrequest.hscic.gov.uk/.
