# Supplementary figures and images for: English hospital episode data analysis (1998–2018) reveal that the rise in dog bite hospital admissions is driven by adult cases
Source: Sci Rep. 2021 Jan 19;11:1767. doi: 10.1038/s41598-021-81527-7 (PMC7815787; doi:10.1038/s41598-021-81527-7)

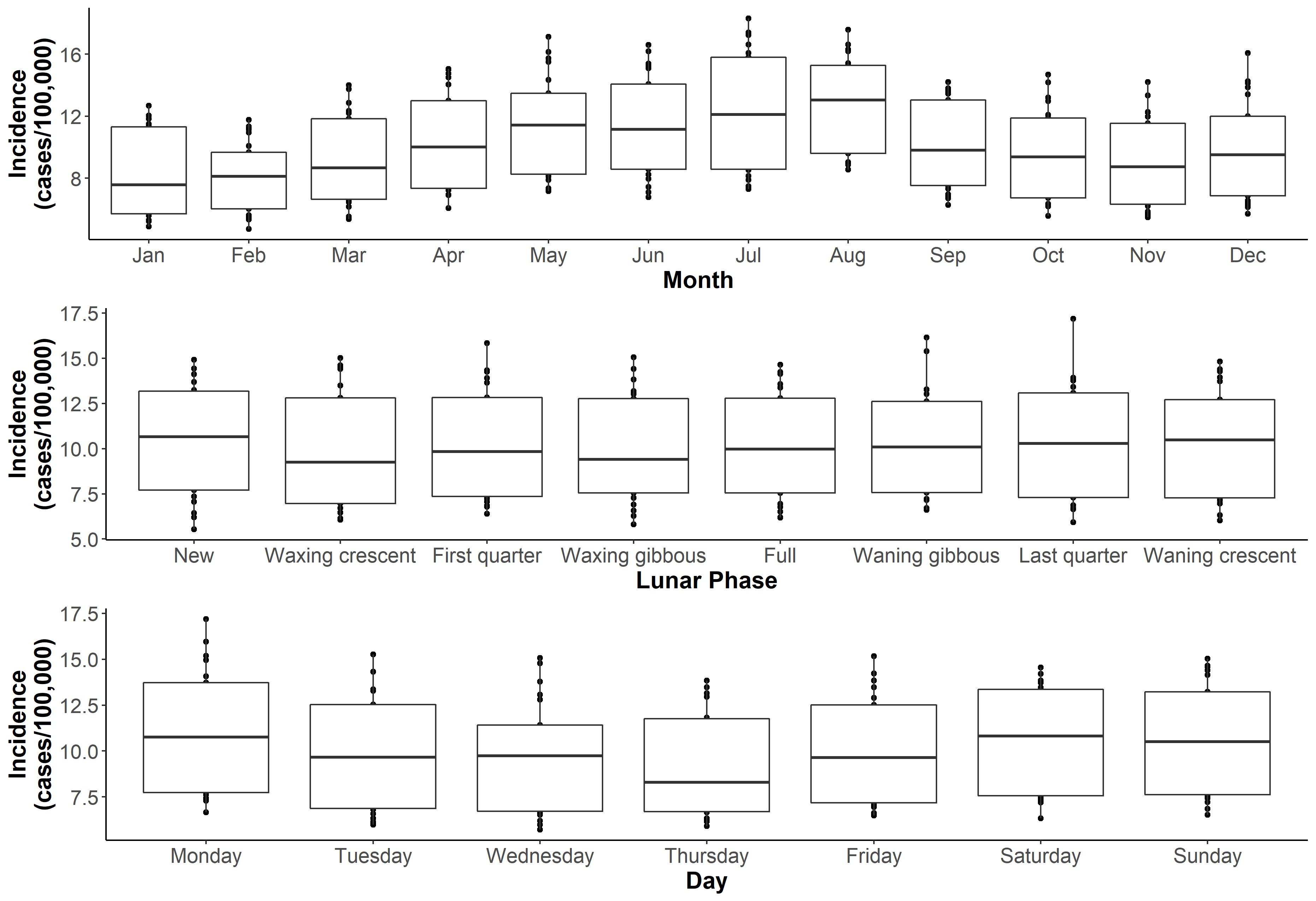

Supplement: Supplementary file 1 — Supplementary Figure S1. [file 41598_2021_81527_MOESM1_ESM.tiff]
