# Supplementary material for: English hospital episode data analysis (1998–2018) reveal that the rise in dog bite hospital admissions is driven by adult cases
Source: Sci Rep. 2021 Jan 19;11:1767. doi: 10.1038/s41598-021-81527-7 (PMC7815787; doi:10.1038/s41598-021-81527-7)
Supplement: Supplementary file 2 — Supplementary Information. [file 41598_2021_81527_MOESM2_ESM.pdf]

Title: English hospital episode data analysis (1998 – 2018) reveal that the rise in dog bite hospital admissions is driven by adult cases

Authors:

John SP Tulloch [1,2,3] (0000-0003-2150-0090) * Corresponding Author (jtulloch@liverpool.ac.uk)

Sara C Owczarczak-Garstecka [3,4,5] (0000-0001-5323-8117)

Kate M Fleming [6] (0000-0002-6572-5016)

Roberto Vivancos [2, 7] (0000-0002-8203-8867)

Carri Westgarth [3] (0000-0003-0471-2761)

Affiliations

1. NIHR Health Protection Research Unit in Emerging and Zoonotic Infections, University of Liverpool, L69 3GL, UK
2. Public Health England, Liverpool, L3 1DS, UK
3. Institute of Infection, Veterinary and Ecological Sciences, University of Liverpool, CH64 7TE, UK
4. Institute of Risk and Uncertainty, University of Liverpool, Liverpool, L69 7ZF, UK.
5. Dogs Trust, London, EC1V 7RQ, UK
6. Institute of Population Health, University of Liverpool, University of Liverpool, L69 3GL, UK
7. NIHR Health Protection Research Unit in Emerging and Zoonotic Infections, Public Health England, Liverpool, L3 1DS, UK

Supplementary Material

Bite setting, resultant injury, and patient management

Methods

A Fisher's exact test was performed to understand if there were child-adult differences in ICD-10 codes that specified the setting of the bite. Based on the ICD-10 code residing in the first 'diagnosis' field, the anatomical location and type of pathological condition resultant of the dog bite were also described and stratified by child-adult status. The day of attendance, month of admission, duration of admittance and treating speciality were analysed descriptively. The route of admission (ie through Accident and Emergency (A&E) departments or from primary care) and discharge destination were described. Further, information from A&E records were collected, these were related to sex, child-adult status, time spent in A&E, and the specific hospital.

Results

Where a dog bite setting was recorded, via the ICD-10 codes, the majority of dog bites happened in a 'home'; 80% of adults, 91% of children. There was no significant difference regarding the setting of a bite between adults and children (p=1), (Table S2).

Over a thousand (n=1,280) different ICD-10 codes were identified in the primary diagnosis fields of the dog bite cases. Codes relating to the ICD-10 chapter 'Injury, poisoning and certain other consequence of external causes': S00-T98', represented 89.6% of all adult cases and 97.1% of child cases. The most common injured anatomical location was the wrist and hand in adults (50.2% of all injuries) and the head in children (70.0% of all injuries) (Table S1). The predominant injury type for both adults and children were open wounds, 76.0% and 92.8% of patients respectively.

There was a seasonal pattern with admissions peaking in July (mean incidence of 12.10, 95% CI 10.40-13.80), cases per 100,000 population per month) and August (12.40, 95% CI 11.00-13.80) and cases at their lowest in January (8.41, 95% CI 7.18-9.63) and February (8.00, 95% CI 6.95-9.04) (Supplementary Fig 1). Day of admission were cyclical in nature with Monday being the busiest day of admission (mean

incidence of 11.10, 95% CI 9.57-12.60 cases per 100,000 per day) and the quietest day Thursday (9.42, 95% CI 8.14-10.7). Out of interest, there was no obvious trend regarding lunar phase and dog bite mean incidence. The mean duration of admission was 1.83 days (median=1, range 0-368 days). Thirty-two percent (32.5%, n=36,692) were managed as day cases and 28.8% of cases were admitted for one night. Three percent of cases stayed for more than seven days (3.4%, n=3,818). There were differences in length of stay between adults and children. Adults mean duration of admission was 2.08 days (median=1, range 0-368 days), whilst that in children was 1.08 days (median=1, range 0-63 days). Thirty percent of adults (30.8%) were managed as day cases, 25.1% were admitted for one night and 4.2% were admitted for more than 7 days. Thirty-seven percent of children (37.5%) were managed as day cases, 39.7% were admitted for one night, and 0.7% were admitted for more than 7 days.

Sixty-seven different consultant specialities treated adult hospital admissions. The top five specialities, representing 92.6% of cases, were plastic surgery (47.3%), trauma and orthopaedic (32.4%), accident and emergency (6.3%), general medicine (4.4%) and general surgery (2.2%). Thirty-eight different specialities treated child admissions. The top five specialities, representing 92.5% of cases, were plastic surgery (50.2%), oral surgery (16.1%), paediatrics (10.7%), maxillofacial surgery (8.3%), and trauma and orthopaedics (7.2%).

The majority of dog bite admissions for both adults and children arrived through accident and emergency departments and the majority of patients were discharged directly to their place of residence (Table S2). Eighty-three deaths in hospital due to dog bites were recorded in admission records; none of these were children. Of A&E records where sex was known (n=5,739), 54.3% were male and 45.4% were female. Of those with age data (n=5,620), 17.7% were classified as children. The median time spent in A&E was 109 minutes (range: 4-589).

Discussion

Many studies describe the majority of dog bites occurring at home [36,38,42,71]. Despite there being no overall statistical difference between the settings in which adult and children's bites occur, adults

received more than double the proportion of bites when on the street (15.3%) than children (6.4%). Patients bitten on the street could be associated with the high number of delivery workers who encounter unknown dogs during their working days [29]. As we have limited information in regards to the bite context, we can draw no conclusions about whether familiarity with the dog [11,14,16,72] or dog breed [11,36,45,69] pose differing risks. The seasonality data matches previous work that identifies peaks in summer months [29,40,71,72] and at weekends and Mondays compared to low incidences on Thursdays [8]. Due to the time period and number of patients studied we hope that this study puts to rest the myth linking dog bites and full moons [73,74]. Our data concur with the wider literature that adults mainly receive injuries to the extremities of the limbs and children to the head [7,10,33,36,38,45]. The large number of ICD-10 codes identified highlight the breadth of the type of injuries that can be sustained resultant of a dog bite.

Figure Legend

Suppementary Material, Figure 1 - Seasonality of English dog bite hospital admission by month, lunar phase and day of the week

Table S1. The anatomical location and injury type resultant of a dog bite in English hospital admissions data. (Blue columns represent adults; green columns represent children)

Anatomical location / Injury Type	Total		Abdomen, lower back, lumbar spine and pelvis		Ankle and foot		Genitals		Head		Hip and thigh		Knee and lower leg		Neck	
Open wounds	76.0%	92.78%	0.31%	0.60%	1.36%	0.78%	0.48%	0.77%	14.20%	70.03%	1.47%	1.59%	5.81%	3.06%	0.17%	0.47%
Fracture	13.2%	2.39%	0.21%	0%	0.64%	0.05%	0%	0%	0.44%	0.38%	1.60%	0.38%	3.71%	0.34%	0.02%	0%
Muscle/tendon injury	3.1%	0.56%	0.01%	0%	0.05%	0.02%	0%	0%	0.03%	0.09%	0.08%	0.01%	0.18%	0.05%	0%	0%
Traumatic Amputation	2.9%	0.47%	0%	0%	0.03%	0.03%	0.01%	0%	0.18%	0.19%	0%	0%	0%	0%	0%	0%
Superficial injury	1.5%	1.76%	0.11%	0.10%	0.03%	0.03%	0%	0%	0.25%	1.26%	0.09%	0.05%	0.35%	0.05%	0.1%	0.01%
Nerve injury	1.3%	0.23%	0%	0%	0%	0%	0%	0%	0.01%	0.05%	0%	0%	0.01%	0.01%	0%	0%
Sprain	0.3%	0.01%	0%	0%	0.01%	0%	0%	0%	0%	0%	0%	0%	0.1%	0%	0%	0%
Contusion	0.3%	0.18%	0.02%	0.01%	0%	0.01%	0.02%	0.02%	0.02%	0.07%	0.03%	0%	0.1%	0.01%	0%	0%
Blood vessel injury	0.2%	0.05%	0%	0%	0%	0%	0%	0%	0%	0.01%	0.01%	0%	0.01%	0.01%	0%	0%
Dislocation	0.2%	0.05%	0%	0%	0.01%	0%	0%	0%	0.01%	0.02%	0%	0%	0.03%	0%	0%	0%
Crushing Injury	0%	0%	0%	0%	0%	0%	0%	0%	0%	0%	0%	0%	0%	0%	0%	0%
Unspecified	0.7%	1.01%	0.09%	0.08%	0.01%	0%	0%	0%	0.22%	0.78%	0.04%	0.01%	0.09%	0.02%	0%	0%
Other	0.2%	0.51%	0.02%	0.01%	0%	0%	0%	0%	0.21%	0.43%	0%	0%	0%	0%	0%	0%
Total	74311	27696	0.77%	0.81%	2.15%	0.93%	0.50%	0.79%	15.57%	73.32%	3.32%	2.05%	10.38%	3.56%	0.21%	0.48%

Anatomical location / Injury Type	Shoulder and upper arm		Thorax		Wrist and hand		Multiple body regions		Unspecified	
Open wounds	3.38%	2.24%	0.19%	0.45%	38.54%	8.10%	0.17%	0.54%	0.38%	0.88%
Fracture	0.32%	0.14%	0.07%	0.02%	5.06%	0.60%	0%	0%	0%	0%
Muscle/tendon injury	0.13%	0.07%	0%	0%	1.75%	0.22%	0%	0%	0.01%	0%
Traumatic Amputation	0%	0%	0%	0%	2.66%	0.25%	0%	0%	0%	0%
Superficial injury	0.07%	0.04%	0.01%	0.02%	0.42%	0.15%	0.03%	0.01%	0.03%	0.01%
Nerve injury	0.01%	0%	0%	0%	1.15%	0.15%	0%	0%	0%	0%
Sprain	0%	0%	0%	0%	0.16%	0%	0%	0%	0%	0%
Contusion	0.01%	0.01%	0.01%	0%	0.04%	0.04%	0%	0%	0%	0%
Blood vessel injury	0.03%	0.01%	0%	0%	0.11%	0.02%	0%	0%	0%	0%
Dislocation	0.04%	0%	0%	0%	0.08%	0.01%	0%	0%	0%	0%
Crushing Injury	0%	0%	0%	0%	0.01%	0%	0%	0%	0%	0%
Unspecified	0.01%	0.01%	0.01%	0%	0.22%	0.06%	0%	0%	0.01%	0%
Other	0%	0%	0.01%	0%	0%	0.01%	0%	0%	0%	0.04%
Total	4.00%	2.53%	0.31%	0.49%	50.21%	9.63	0.21%	0.55%	0.43%	0.94%

Supplementary Material, Table S2. The admission source and discharge destination of dog bite hospital admissions

Admission Source	Adults (n=84,020)	Children (n=28,621)	Discharge Destination	Adults (n=84,020)	Children (n=28,261)
Accident and Emergency	78.4%	81.0%	Home	91.5%	96.8%
Primary Care	3.4%	3.4%	Hospital Care	1.6%	1.2%
Elective	15.2%	9.9%	Death	0.1%	0%
Other	2.9%	5.7%	Other	0.3%	0.1%
			Unknown	6.5%	1.9%
